# Gender differences in metformin effect on aging, life span and spontaneous tumorigenesis in 129/Sv mice

**DOI:** 10.18632/aging.100245

**Published:** 2010-12-14

**Authors:** Vladimir N. Anisimov, Tatiana S. Piskunova, Irina G. Popovich, Mark A. Zabezhinski, Margarita L. Tyndyk, Peter A. Egormin, Maria N. Yurova, Svetlana V. Rosenfeld, Anna V. Semenchenko, Irina G. Kovalenko, Tatiana E. Poroshina, Lev M. Berstein

**Affiliations:** N.N.Petrov Research Institute of Oncology, Pesochny-2, St.Petersburg 197758, Russia

**Keywords:** metformin, biomarkers of aging, life extension, carcinogenesis, mice

## Abstract

Studies in mammals have led to the suggestion that hyperglycemia and hyperinsulinemia are important factors both in aging and in the development of cancer. It is possible that the life-prolonging effects of calorie restriction are due to decreasing IGF-1 levels. A search of pharmacological modulators of insulin/IGF-1 signaling pathway (which mimetic effects of life span extending mutations or calorie restriction) could be a perspective direction in regulation of longevity. Antidiabetic biguanides are most promising among them. The chronic treatment of inbred 129/Sv mice with metformin (100 mg/kg in drinking water) slightly modified the food consumption but failed to influence the dynamics of body weight, decreased by 13.4% the mean life span of male mice and slightly increased the mean life span of female mice (by 4.4%). The treatment with metformin failed influence spontaneous tumor incidence in male 129/Sv mice, decreased by 3.5 times the incidence of malignant neoplasms in female mice while somewhat stimulated formation of benign vascular tumors in the latter.

## INTRODUCTION

The link between aging and insulin/IGF-1 signaling has attracted substantial attention during last years. The potential connection was evidenced by an increase in incidence of insulin resistance and type 2 diabetes in accelerated aging syndromes, on the one side, as well as by life span extension due to caloric restriction (CR) in rodents, on the other[1]. Concomitant reduction in plasma insulin and plasma glucose levels, which implies increased sensitivity to insulin, emerges as a hallmark of increased longevity [2]. Hyperglycemia is an important aging factor involved in generation of advanced glycosylation endproducts (AGEs) [3,4]. There is evidence that hyperinsulinemia favors accumulation of oxidized protein by reducing its degradation as well as facilitates protein oxidation by increasing steady-state level of oxidative stress [3]. Untreated diabetics with elevated glucose levels suffer many manifestations of accelerated aging, such as impaired wound healing, obesity, cataracts, vascular and microvascular damage [5]. It is important to stress that hyperinsulinemia is an significant factor not only in aging but also in the development of cancer [5,13-18].

The concept of CR mimetics is now being intensively explored [9-12]. CR mimetics involve interventions that produce physiological and anti-aging effects similar to CR. It was suggested to use biguanide antidiabetics as a potential anti-aging treatment [5,13-18]. The anti-diabetic drugs, phenformin and buformin, were observed to reduce hyperglycemia and produce the following effects: improved glucose utilization; reduced free fatty acid utilization, gluconeogenesis, serum lipids, insulin and IGF-1, and reduced body weight both in humans and experimental animals [5,16-19]. The use of phenformin in humans has been limited the last two decades because of a potential association with lactic acidosis. Widely used antidiabetic biguanide, metformin (dimethylbiguanide) does not increase risk for lactic acidosis or for increased lactate levels in type 2 diabetes [20] but has some adverse effects, including renal insufficiency in some patients [21], vitamin B_12_ deficiency [22], and gastrointestinal disturbances [23]. In transgenic female HER-2/neu mice, it was shown that metformin slowed down aging and tumor development [24,25]. However, in female SHR mice metformin significantly increased life span but failed to inhibit spontaneous tumorigenesis [26]. Recently it was shown that treatment with metformin did not influence life span of male F344 rats [27]. To test strain and gender factors in present study we evaluated effects of metformin on some parameters of aging, life span and spontaneous tumorigenesis in inbred male and female 129/Sv mice.

## RESULTS

### Age-related body weight dynamics

**Figure 1.**
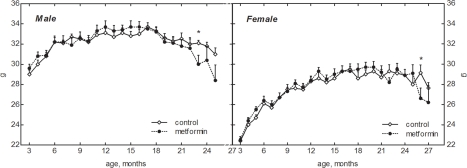
Dynamics of body weight in male and female 129/Sv mice treated or non-treated with metformin

Male 129/Sv mice were heavier than females over the life span both in the control and treated with metformin groups (Fig. 1). The body weight of mice in both control and metformin-treated groups increased with age, exceeding at 18 months the body weight of 3-month-old animals by 27.7% in the control female group, and by 30.5 % in the female group treated with metformin. The corresponding indices for males were 14.8% and 12.2%. Thereafter the body weight decreased both in male and female mice. On the other hand, there was no difference in the mean body weight of mice exposed and non-exposed to the drug until the age of 22 months while afterwards a tendency to the decrease of body weight was observed in metformin treated group (Fig. 1).

### Age-related dynamics of food and water consumption

**Figure 2.**
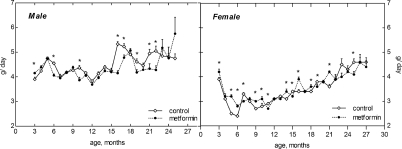
Dynamics of food consumption in male and female 129/Sv mice treated or non-treated with metformin

**Figure 3.**
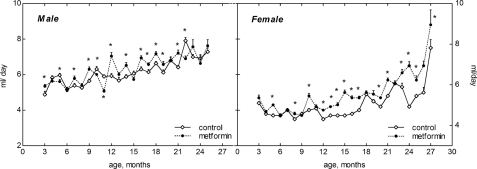
Dynamics of drinking water consumption in male and female 129/Sv mice treated or non-reated with metformin

Food consumption gradually increased after the age of 15 months both in female and male mice. The amount of food daily consumed by male mice was higher than in females throughout the whole period of observation (Fig. 2). It was approximately similar in the controls and in metformin-treated male and female groups until the age of 1 year. However, at the age of 15 to 21 months the food consumption was slightly but significantly (p<0.05) decreased in metformin-treated male group as compared with respective control (Fig. 2). The amount of water consumed by mice varied during the period of observation and was slightly increased by 10-20% in metformin-treated group practically at all ages (Fig. 3). Again (like in case of food consumption), males drank more water than females in both control and metformin groups.

### Age-related dynamics of body temperature

**Figure 4.**
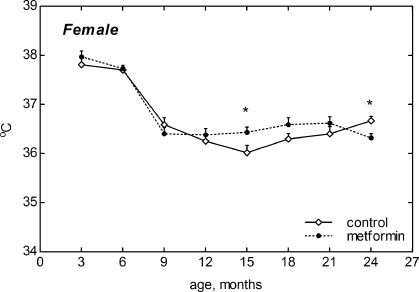
Dynamics of body temperature in female 129/Sv mice treated or non-treated with metformin

The body temperature was measured only in females. It decreased between 6th and 9th month of life and was practically at the same level until the age of 24 months. Temperature was higher in metformin-treated group than that in the controls at the age of 15 month of age but was lower than in control group at the age of 24 months (Fig. 4).

### Age-related dynamics of estrous function in mice

The length of estrous cycles in the control and metformin-treated female 129/Sv mice was not significantly changed with age, whereas it was shorter in the group of animals treated with metformin and aged 6 and 9 months (Table 1). The fraction of mice with regular estrous cycles decreased with the age uniformly in the control and metformin-treated groups (Table 1).

**Table 1.** Effect of metformin on age-related dynamics of estrous functional parameters in female 129/Sv mice

Age, months	Length of estrous cycle (days)	Rate of estrous cycles of various length (%)			Fraction of mice with regular cycles (%)
		<5 days	5-7 days	>7 days	
*Control*					
3	5.8 ± 0.25	24	58	18	83
6	5.6 ± 0.24	26	61	13	92
9	6.0 ± 0.24	11	78	11	98
12	5.3 ± 0.28	36	50	14	94
15	4.8 ± 0.20	43	51	6	93
18	5.6 ± 0.25	27	58	15	88
21	6.1 ± 0.38	18	64	18	75
24	7.0 ± 0.79	0	78	22	62
*Metformin*					
3	5.9 ± 0.22	13	81*	6	93
6	4.9 ± 0.17*	42	53	5	100
9	5.3 ± 0.25*	37**	51**	12	95
12	5.2 ± 0.18	37	59	5	98
15	5.1 ± 0.28	28	70	2	93
18	5.5 ± 0.25	12	82*	6	87
21	5.9 ± 0.45	14	68	18	72
24	6.2 ± 0.62	20	50	30	52

Notes: Difference with controls of corresponding age in the control group is significant:

* p<0,05;

** p<0,01.

**Table 2.** Effect of treatment with metformin on metabolic parameters in the serum of 21-months-old male 129/Sv mice

Group	Glucose, mM/l	Total cholesterol, mM/l	Triglycerides, mM/l	Insulin, mkU/ml
Control	5.18 ± 0.30	3.44 ± 0.13	1.32 ± 0.07	1.64 ± 0.30
Metformin	6.14 ± 0.47	3.13 ± 0.12	1.30 ± 0.57	1.35 ± 0.08

There were 10 animals in each group.

### Metabolic and hormonal parameters in mice treated and non-treated with metformin

No any difference was revealed in the levels of glucose, total cholesterol, triglycerides and insulin between 21-month-old male mice in control and metformin-treated group (Table 2).

**Table 3.** Effect of metformin on the age-related dynamics of the chromosome aberrations (ChA) incidence in bone marrow cells in male 129/Sv mice

Age, months	Treatment	Total incidenceof ChA, %	Single bridges, %	Multiple bridges, %	Fragments, %
4	Control	15.4 ± 0.02	8.1 ± 0.01	5.3 ± 0.02	2.0 ± 0.01
7	Control	19.1 ± 0.01	8.0 ± 0.01	11.1 ± 0.01	0
	Metformin	27.3 ± 0.01*	14.1± 0.02*	12.1 ± 0.01	1.1 ± 0.01
18	Control	23.0 ± 0.06	7.2 ± 0.02	15.30 ± 0.04	0.5 ± 0.01
	Metformin	28.0 ± 0.05*	15.1±0.02 *	12.50 ± 0.02	0.4 ± 0.01
22	Control	28.9 ± 0.02	8.5 ± 0.01	18.0 ± 0.02	2.4±0.01
	Metformin	34.8± 0.03*	20.3± 0.06*	12.2 ± 0.03*	2.3 ± 0.01

The difference with the control of the same age is significant:

* - p<0,001

The total incidence of the chromosome aberrations in bone marrow of both control and treated mice increased with age. As compared with controls the chromosome aberrations value was increased in mice treated with metformin at the age 7, 18 and 22 months (p<0.001) (Table 3). Of note, this increase was related to the increase of incidence of single bridges whereas the incidence of multiple bridges was significantly decreased in 22-month-old metfromin-treated mice as compared with control mice of the same age.

### Survival and longevity of 129/Sv mice

**Figure 5.**
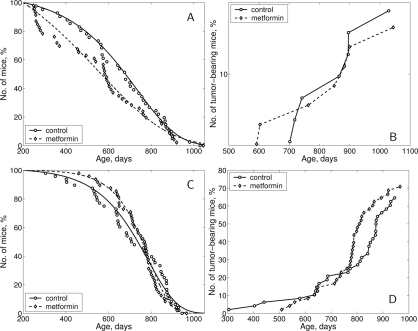
Survival curves and tumor yield curves of 129/Sv mice treated or non-treated with metformin (**A**) - survival curves, males; (**B**) - tumor yield curves, males; (**C**) - survival curves, females; D - tumor yield curves, females.

According to the log-rank test [28] the difference in survival of both male and female 129/Sv mice exposed to metformin treatment, compared to the control group, is non-significant (p-value is 0.359 for males and 0.652 for females). Survival dynamics in control and metformin-treated male and female mice are shown in Fig. 5, A and C. It is worthy of note that though there is no difference in life span distributions between male and female controls (p-value is 0.571, χ2 = 0.3 on 1 df) metformin treatment evidently produced somewhat different effect on survival of male and female mice, since the difference in life span distributions of males and females under metformin action became statistically significant (p-value is 0.0474, χ^2^ = 3.9 on 1 df).

In males, metformin treatment significantly decreased the mean life span of all mice (−13.4%, p < 0.05) but not so in long-living individuals (last 10% of survivors) as well as it did not influence maximum life span (Table 4). In females, the mean life span and median of this parameter in metformin-treated animals were slightly increased (+4.4% and 7.8% respectively, p>0.05) in comparison to the controls, whereas mean life span of last 10% survivors and maximum life span were the same as in the control (Table 5). However, it deserves mentioning that until the age of 700 days survived 54.1% of mice in the control female group and 72.9% of metformin-treated females (p<0.03; Fischer's exact test).

Parameter α of the Gompertz model, which is interpreted as the rate of aging, was lower (by 1.67 times) in the male group subjected to metformin treatment than in controls males, whereas the mortality rate doubling time (MRDT) was increased in metformin-treated group of male mice (Table 4). In females, the parameter α was increased by 1.43 times and MRDT was decreased as compared to the relevant controls (Table 5).

According to the estimated parameters of the Cox's regression model being a female under metformin treatment decreases the relative risk of death compared to the male group kept under the same treatment. Cox's regression model parameters for all mice (males and females) was estimated as follows: β = −0.415; exp(β) = 0.66; se(β) = 0.211; p = 0.0504.

**Table 4.** Effect of metformin on life span and tumorigenesis in male 129/Sv mice

Parameters	Control	Metformin
Number of mice	41	46
Effective number of mice	25	22
Mean life span, days (M±S.E.M.)	662±30.0	573±33.7* (−13.4%)
Mediana, days	680	586
Mean life span of last 10% survivors, days	951±32.5	931±28.7
Maximum life span, days	1029	1044
α×10^−3^, days^−1^	5.19 (4.37; 6.27)	3.10 (2.42; 3.48)*
MRDT, days	134 (111; 159)	224 (199; 287)*
Number of tumor-bearing mice (%)	7 (28.0%)	7 (31.8%)
Number of malignant tumor-bearing mice (%)	5 (20.0%)	5 (22.7%)
Mean life span of tumor-bearing mice, days	835±45.2	804±62.1
Total number of tumors	11	8
Number of malignant tumors	6	5
*Localization and type of tumors*		
Skin: carcinoma	1	-
Liver: haemangioma	1	1
hepatocellular carcinoma	2	1
Lung: adenoma	- 3	2 2
adenocarcinoma	3	2
Spleen: leukemia	-	1
Colon: polyp	1	-
adenocarcinoma	-	1
Prepucial gland: cystadenoma	2	-
Harderian gland: cystadenoma	1	-

Notes: Difference with the controls is significant:

* - p<0,05.

α - aging rate; MRDT - mortality rate doubling time, days (in brackets - 95% confidential interval)

### Spontaneous tumor development in 129/Sv mice

The dynamics of age-related increase in spontaneous tumor development is represented at the Fig. 5, B and D. The mean latent period of tumor development was similar in male and female mice of both groups (Tables 4 and 5). According to the long-rank test [28] there were no significant differences in age-related distributions of the total tumors occurrence in control and metformin-treated groups of both sexes.

The first tumor-bearing male mouse died at the age of 592 days in the metformin-treated group and at the 702 days in the control male group. Total tumor incidence in effective control male mice (survived by the time of the death from the first tumor in the experiment) was 28.0% and 31.8% in the metfromin-treated animals (Table 4). There were no significant differences in the incidence of malignant or benign tumors of any localization between the control and metformin-treated male mice.

In the female mice, the total tumor incidence was similar in the control and metformin-treated groups. However, the incidence of malignant tumors was significantly decreased (by 3.5 times) in the group given metformin in comparison to the control (Table 5). Benign vascular tumors of the uterus and ovary developed most frequently in female 129/Sv mice (Table 5), in line with oncological characteristics of this strain of mice [29], and were discovered more often in metformin-exposed animals. Thus, *in toto* uterine and ovarian haemangiomas and haemangioendoteliomas were revealed respectively in 54.2% of control and 68.8% of metformin-exposed female mice, p < 0,03 (Fisher's exact test). At the same time the differences in the incidence of angiogenic tumors only of ovaries or utery between treated and untreated with metformin was not statistically significant.

There was no significant difference in the incidence of any other tumors between mice treated or not with metformin.

**Table 5.** Effect of metformin on life span and tumorigenesis in female 129/Sv mice

Parameters	Control	Metformin
Number of mice	48	48
Effective number of mice	48	48
Mean life span, days (M±S.E.M.)	711± 24.3	742 ± 15.8 (+4.4%)
Median, days	715	771 (+7.8%)
Mean life span of last 10% survivors, days	910 ± 8.9	913 ± 19.2
Maximum life span, days	945	966
α×10^−3^, days^−1^	7.27 (6.36; 8.20)	10.40 (8.90;12.50)*
MRDT, days	95 (84;109)	67 (56; 78)*
Number of tumor-bearing mice (%)	31 (64.6%)	34 (70.8%)
Number of malignant tumor-bearing mice (%)	7 (15.6%)	2 (4.2%)*
Mean life span of tumor-bearing mice, days	766 ± 27.3	764 ± 18.4
Total number of tumors	46	50
Number of malignant tumors	9	2*
*Localization and type of tumors*		
Uterus: haemangioma & haemangioendotelioma adenocarcinoma sarcoma	23 2 1	30 1 -
Ovary: granulesa-cell tumor haemangioma & haemangioendothelioma cystadenoma	3 7 2	1 11 5
Lung: adenoma adenocarcinoma	2 4	1 1
Liver: hepatocellular carcinoma	1	-
Haematopoietic tissue: leukemia	1	-

Note: Difference with the control is significant:

* - p<0,005.

α - aging rate; MRDT - mortality rate doubling time, days (in brackets - 95% confidential interval).

## DISCUSSION

In this paper for the first time were shown gender differences in effect of metformin on survival, life span and spontaneous carcinogenesis in 129/Sv mice. The reasons for these differences are at present unclear. There are several possibilities explaining these observations. One of them could be fundamental differences in mechanism of aging in males and females, another - gender peculiarities in the targets of drugs, including metformin. This question needs a special discussion.

**Table 6.** Effect of antidiabetic drugs on life span and spontaneous carcinogenesis in rodents

Species	Strain	Sex	Drug	Effect on mean life span	Effect on maximum life span	Effect on spontaneoustumor development^c^	References
Mice	C3H/Sn	F	Phenformin	+21.8%	+26.0%	↓	[13]
	FVB/N^a^	F	Metformin	+8.0%	+16.2%	↓	[24]
	FVB/N^a^	F	Metformin	+6.7%	-19.3%	↓	[25]
	SHR	F	Metformin	+37.9%	+10.3%	=	[26]
	NMRI	F	Diabenol	+6.7%	+1.4%	↓	[30]
	FVB/N^a^	F	Diabenol	0	+6.0%	=	[30]
	HD^b^	M	Metformin	+20.1%	+18.5%	N.D.^d^	[31]
	HD^b^	F	Metformin	0	0	N.D.^d^	[31]
	129/Sv	F	Metformin	+4.4%	+7.8%	↓ malignant. ↑ benign	Present paper
	129/Sv	M	Metformin	-13.4%	+1.5%	=	Present paper
Rat	LIO	F	Phenformin	0	9.8%	↓	[15]
	LIO	F	Buformin	+7.3%	+5.5%	↓	[15]
	F344	M	Metformin	+2.4%	0	N.D.^d^	[27]

Note:

a FVB/N mice transgenic HER-2/neu gene;

b HD, transgenic mouse model of Huntington's disease (R6/2 line with ∼ 150 glutamine repeats);

c ↓ Decrease in incidence and/or increase in tumor latency; = no effect;

d No data.

Several years ago, it was originally suggested to use antidiabetic biguanides as mimetics of caloric restriction (CR) and a potential anti-aging treatment [5]. In a number of studies it was shown that treatment with biguanides (phenformin, buformin and metformin) increased life span and suppressed spontaneous tumorigenesis, however, in some experiments it failed to modify these parameters (Table 6). The causes of the observed contradictions are not completely clear. One of them could be differences in a dose of the drug, however, it is worthy of note that male and female 129/Sv mice demonstrated different reaction to the same dose of metformin. Also, it deserves mentioning that metformin failed to increase life span of male F344 rats [27].

Inhibitor of mammalian target of rapamycin (mTOR) increased life span of female in larger extend than in male hybrid mice [33]. It is worthy to note, the mean life span extension was observed in female (+20.4%) but it was not increased in male *S6K1^−/−^*mice [34]. Deletion of ribosomal S6 protein kinase 1 (*S6K1*), a component of the nutrient-responsive mTOR signaling pathway, led to increased life span in mice and to motor dysfunction and loss of insulin sensitivity [34]. Deletion of *S6K1* induced gene expression patterns similar to those seen in CR or with pharmacological activation of adenosine monophosphate (AMP)-activated protein kinase (AMPK), a conserved regulator of the metabolic response to CR. The mean life span of oldest 10% survivors and maximum life span were also increased only in females. There was no difference in the incidence of macroscopic tumors in *S6K1^−/−^* and wild type mice.

Comparison of effects of metformin on various biomarkers of aging in different strains of mice shows rather notable similarity in observed patterns (Table 7). Thus, the body weight and food consumption were unchanged or slightly decreased at some periods of life in metformin-treated rodents. The body temperature was similar in control and metformin-treated mice. In females of 3 strains (129/Sv, HER-2/neu and SHR), the slow down of age-related disturbances in estrous function have been observed. It is worthy of note that in women with polycystic ovary syndrome metformin improves menstrual regularity, leading to spontaneous ovulation, and enhances the induction of ovulation with clomiphene citrate [35].

**Table 7.** Effect of metformin on biomarkers of aging in rodents of various strains

Strain, species:	129/Sv mice	SHR mice	HER-2 mice	F344 rats
Sex:	M	F	F	F	F	M
References:	Present paper	[26]	[24]	[25]	[27]
Body weight	↓	=	=↓	=	↓	=
Food consumption	↓	=	=	↓	=	=
Body temperature	ND	↑	=	=	=	=
Estrous function	↑		↑	↑	=	
Tumor development	=	↓ malignant ↑ benign	=	↓	ND	ND
Glucose	=	ND	=	↓	=	ND
Triglycerides	=	ND	↓	↓	ND	ND
Cholesterol	=	ND	=	=	ND	ND
Insulin	=	ND	=	=	=	ND
Life span	↓	↑	↑	↑	=	=

Notes: ND - not detected; ↓ - decreased; ↑ - increases; = no effect.

Treatment with metformin failed influence metabolic parameters in male 129/Sv mice and was followed by some improvements in female SHR and HER-2/neu mice. Metformin inhibited tumorigenesis in female 129/Sv and HER-2/neu mice and did not affect it in male 129/Sv and female SHR mice. In female 129/Sv mice treatment with metformin inhibited development of malignant tumors and increased the total incidence of benign angiogenic tumors. These data are in according with observations that metformin can to increase VEGF expression, intratumoral microvascular density and reduced necrosis thus promote the angiogenic phenotype and increased tumorigenic progression [36].

The incidence of chromosome aberrations in male 129/Sv mice was significantly higher at young, middle and old age than that in male CBA, SHR, FVB/N, C57BL/6J mice of the same age groups [37]. Treatment with metformin increased the incidence of chromosome aberrations in male 129/Sv mice that was in agreement with the observed reduction of life span in males exposed to the drug. The comparative study on the effect of metformin on female and male F344 rats would be important for understanding gender differences in response to metformin in rodents.

It is worthy to note, that experiments in yeast and *C.elegans* show that the life extension by CR is not a mechanical output of low calories and consequence of a reduction in ROS or AGE formation, but a process that is highly regulated, triggering metabolic shift toward respiration that activates the regulator SIR2 [38]. It was observed that phenformin inhibits proliferation and induced enhanced and transient expression of the cell cycle inhibitor p21 and apoptosis in human tumor cells lines [39].

Buformin was supplemented to nutrient medium in various concentrations (from 1.0 to 0.00001 mg/ml) during the larvae stage and over the life span of *C. elegans*. The drug given at the concentration of 0.1 mg/ml increased the mean life span of the worms by 23.4% (p < 0.05) and the maximum life span by 26.1% as compared to the controls [40]. Metformin supplementation (50 mM dose) was shown to increase the mean life span, but not maximum, of *C. elegans*, although 10 or 100 mM doses showed no significant life span benefit [41]. The authors have shown that metformin prolongs nematode healthspan, slowing lipofuscin accumulation, extending mean life span, and prolonging youthful locomotor ability in a dose-dependent manner. Genetic data suggest the metformin acts through a mechanism similar to that operative in eating-impaired CR mutants, but independent of insulin signaling pathway. Energy sensor AMPK and AMPK-activating kinase LKB1, which are activated in mammals by metformin treatment [42,43], are essential for health benefits in *C. elegans,* suggesting that metformin engages a metabolic loop conserved across phyla. It was also shown that metformin activated SKN-1/Nrf2, oxidative stress-responsive transcription factor [41].

Thus, biguanides could be potent geroprotectors and anticarcinogens. There are several suggestions on the mechanism of anti-carcinogenic and anti-tumor effects of metformin. Firstly, metformin is commonly considered to function as a sensitizer to insulin [5,43-46]. The antidiabetics biguanides inhibit fatty acid oxidation, suppress gluconeogenesis in the liver, increase the availability of insulin receptors, inhibit monoamine oxidase [44], increase sensitivity of hypothalamo-pituitary complex to negative feedback inhibition, reduce excretion of glucocorticoid metabolites and dehydroepiandrosterone-sulfate [5]. It was shown that metformin decreases platelet superoxide anion production in diabetic patients [47]. The capacity of metformin to activate AMPK (AMP-activated protein kinase) has been suggested to constitute an insulin-independent mechanism of inhibitory effect of metformin on proliferation of cancer cells, directly connected with promotion of the inhibition of the AMPK down-stream mammalian effector target of rapamycin (mTOR) [48-53].

mTOR controls cell growth and metabolism in response to nutrients (e.g., amino acids), insulin and growth factors such as IGF-1. Since TOR is activated by nutrients and insulin, calorie restriction deactivates TOR. Calorie restriction extends life span in a variety of species including primates [12,54]. Recently it was demonstrated that rapamycin like metformin extends life span in mice [33] and delays the development of mammary carcinomas in HER-2/neu transgenic mice [25]. Both rapamycin and biguanides inhibit carcinogenesis in rodents [25,46,55-59].

Our results alongside with recent findings of mTOR signaling pathway involvement in regulation of aging [34] and evidence of significant life span extension of mammals with rapamycin and calorie restriction [33,54] suggest that mimetic of calorie restriction, antidiabetic biguanide metformin may be rapidly contemplated for pharmacological intervention at a population level.

## MATERIALS AND METHODS

### Animals.

Inbred 129/Sv mice were breed at the animal facility of N.N. Petrov Research Institute of Oncology. The mice were kept 5-7 in polypropylene cages (30 x 21 x 10 cm) under standard light/dark regimen (12 hours light :12 hours darkness) at 22 ± 2 °C, and received standard laboratory chow [60] and tap water *ad libitum*.

### Experimental design.

Eighty seven male and 96 female 129/Sv mice at the age of 3 months were randomly divided into two groups. Mice of the first group were given metformin (1,1- Dimethylbiguanide hydrochloride, Biomedicals, France) with drinking water (100 mg/kg) daily, whereas the mice of the second group were given tap water without metformin and served as a control. This dose of metformin is similar to used in our earlier experiments with transgenic HER-2/neu and outbred SHR mice [24-26] and equal to 300 mg/m^2^ of the surface area. Recalculation for humans gives in average 510 mg/m^2^, that much less than commonly used in clinical practice (1.0 - 2.5 g per day). Once a week all mice were palpated for detection of tumor mass appearance. Once a month all mice were weighted and, simultaneously, the amount of consumed food and water was measured, and the rate of the consumed water (ml) and food (g) per mouse were calculated. Once in every 3 months, daily for 2 weeks vaginal smears of the animals were cytologically examined to estimate the estrous function. In the same period, rectal body temperatures of female mice were measured with an electronic thermometer, TPEM (KMIZ, Russia).

At the age of 21 month 10 male mice from the control group and the group treated with metformin were sacrificed by decapitation after overnight starvation. Samples of serum were obtained and stored at the −20°C for subsequent analyses. Other animals were observed until their natural deaths. The date of each death was registered, and the mean life span, the age at which 90% of the animals died, and the maximum life span were estimated.

### Metabolic and hormonal assays.

The serum levels of glucose were estimated by enzymocolorimetric (glucose-oxidase) method with kits from “Impact” (Moscow, Russia); cholesterol and triglycerides - by enzymocolorimetric method with kits of “Olvex” (St.Petersburg, Russia); insulin - by immune enzyme assay (ELISA) with kits from Diagnostic Systems Laboratories, Inc. (U.S.A.).

### Cytogenetic study.

Chromosomal aberrations in bone marrow cells was studied by modified Ford's method described in Rosenfeld et al.[37]. Mice were sacrificed with ether anaesthesia. Both femurs of each mouse were dissected and bone marrow cells flushed gently with 0.56% KCl solution into a centrifuge tube. Cells were treated for 20 min with hypotonic solution and fixed with ethanol: acetic acid mixture (3:1). Slides were stained with 4% acetoorseine. 20-30 well spread anaphases were analyzed for each animal and cells with chromosome breaks, acentric fragments, and other aberrations were evaluated on 1,000X magnification with a light microscope (Leitz, Germany).

### Pathomorphological examination.

All animals were autopsied. Site, number and size of mammary tumors and their metastases in lungs were checked. All tumors, as well as the tissues and organs with suspected tumor development were excised and fixed in 10% neutral formalin. After the routine histological processing the tissues were embedded into paraffin. 5-7 μm thin histological sections were stained with haematoxylin and eosine and were microscopically examined. Tumors were classified according to International Agency for Research on Cancer recommendations [61].

### Statistics.

Experimental results were statistically processed by the methods of variation statistics with the use of STATGRAPH statistic program kit. The significance of the discrepancies was defined according to the Student *t*-criterion, Fischer exact method, χ^2^, non-parametric Wilcoxon-Mann-Whitney and Friedman RM Anova on Ranks. Student-Newman-Keuls method was used for all pairwise multiple comparisons. Coefficient of correlation was estimated by Spearman method [62]. Differences in tumor incidence were evaluated by the Mantel-Haenszel log-rank test.

Parameters of Gompertz model were estimated using maximum likelihood method, non-linear optimization procedure [63] and self-written code in 'Matlab'; confidence intervals for the parameters were obtained using the bootstrap method [64].

For experimental group the Cox regression model [65] was used to estimate relative risk of death and tumor development under the treatment compared to the control group: h(t, z) = h_0_(t) exp(zβ), where h(t,z) and h_0_(t) denote the conditional hazard and baseline hazard rates, respectively, β is the unknown parameter for treatment group, and z takes values 0 and 1, being an indicator variable for two samples − the control and treatment group.
